# Characterization of NLRP12 during the Development of Allergic Airway Disease in Mice

**DOI:** 10.1371/journal.pone.0030612

**Published:** 2012-01-23

**Authors:** Irving C. Allen, John D. Lich, Janelle C. Arthur, Corey M. Jania, Reid A. Roberts, Justin B. Callaway, Stephen L. Tilley, Jenny P.-Y. Ting

**Affiliations:** 1 Lineberger Comprehensive Cancer Center, Institute of Inflammatory Diseases and Center for Translational Immunology, The University of North Carolina at Chapel Hill, Chapel Hill, North Carolina, United States of America; 2 Department of Microbiology and Immunology, The University of North Carolina at Chapel Hill, Chapel Hill, North Carolina, United States of America; 3 Division of Pulmonary and Critical Care Medicine, Department of Medicine, The University of North Carolina at Chapel Hill, Chapel Hill, North Carolina, United States of America; National Jewish Health, United States of America

## Abstract

Among the 22 members of the nucleotide binding-domain, leucine rich repeat-containing (NLR) family, less than half have been functionally characterized. Of those that have been well studied, most form caspase-1 activating inflammasomes. NLRP12 is a unique NLR that has been shown to attenuate inflammatory pathways in biochemical assays and mediate the lymph node homing of activated skin dendritic cells in contact hypersensitivity responses. Since the mechanism between these two important observations remains elusive, we further evaluated the contribution of NLRP12 to organ specific adaptive immune responses by focusing on the lung, which, like skin, is exposed to both exogenous and endogenous inflammatory agents. In models of allergic airway inflammation induced by either acute ovalbumin (OVA) exposure or chronic house dust mite (HDM) antigen exposure, *Nlrp12^−/−^* mice displayed subtle differences in eosinophil and monocyte infiltration into the airways. However, the overall development of allergic airway disease and airway function was not significantly altered by NLRP12 deficiency. Together, the combined data suggest that NLRP12 does not play a vital role in regulating Th2 driven airway inflammation using common model systems that are physiologically relevant to human disease. Thus, the allergic airway inflammation models described here should be appropriate for subsequent studies that seek to decipher the contribution of NLRP12 in mediating the host response to agents associated with asthma exacerbation.

## Introduction

NLRP12 (also MONARCH-1/PYPAF7) is a member of the nucleotide binding domain, leucine rich repeats-containing family (NLR) of proteins, which sense pathogens and pathogen products in the cell cytoplasm [1]. The NLR family of proteins has been increasingly associated with various aspects of innate and adaptive immune system regulation, inflammation, and autoimmunity. Several prototypic NLR family members, including CIITA, NLRP3 and NOD2, have emerged as major contributing factors in a variety of human diseases [2]. To date, the majority of NLR studies have focused on a subgroup of NLR family members that are capable of forming a multiprotein complex, termed the inflammasome, with the NLR adaptor protein PYCARD (ASC) and Caspase-1. The inflammasome functions to cleave pro-IL-1β and pro-IL-18 into their active cytokines. NLRs that are associated with this subgroup are inherently proinflammatory and include NLRP3 and NLRC4 (IPAF). In addition to the inflammasome forming NLRs, recent studies have also revealed a second subgroup of NLRs that have anti-inflammatory functions, which dampen overzealous immune responses. The members of this subgroup include NLRP12, NLRX1, NLRC3 and NLRC5 [3], [4], [5], [6], [7]. While the overwhelming majority of studies have focused on the role of the NLRs in mediating the host innate immune response, several recent studies have suggested that select NLRs may also participate in the initiation of the adaptive immune response.

NLRP12 was originally suggested to form an inflammasome with PYCARD [1]. However, more recently, NLRP12 has been characterized as a negative regulator of both canonical and non-canonical NF-κB signaling [5], [6]. NLRP12 was shown to interact with and inhibit the accumulation of hyperphosphorylated IRAK1, downstream of TLR signaling, to attenuate canonical NF-κB signaling [6]. Similarly, NLRP12 associates with NIK in the non-canonical NF-κB pathway, which results in the rapid proteosomal degradation of the kinase [5]. NF-κB regulates a variety of inflammatory pathways that may directly contribute to asthma pathogenesis. However, because of its central role in innate immunity, NF-κB and modulators of NF-κB signaling are more typically associated with modulating the host immune response to agents associated with asthma exacerbation. Prior to this research, only one additional publication has explored the *in vivo* role of NLRP12. In this previous work, NLRP12 was found to attenuate the development of contact hypersensitivity [8]. The underlying mechanism was found to be associated with altered dendritic cell and granulocyte migration in response to chemokine signaling [8]. This finding was consistent with results from human association studies that reported identifying an association between specific mutations in NLRP12 and a subgroup of atopic dermatitis patients [9]. Because contact hypersensitivity and asthma share many of the same immunopathological features, we sought to characterize the *in vivo* contribution of NLRP12 in common mouse models of allergic airway inflammation.

Anti-inflammatory biopharmaceuticals are considered to be a key element in the clinical regulation of innate and allergic airway inflammation in several human lung diseases. Thus, proteins that function as negative regulators of inflammation are of immense clinical and scientific value. NLRP12 has been shown to be a robust inhibitor of various inflammatory pathways and influence the development of contact hypersensitivity. Therefore, we hypothesized that we would observe attenuated allergic lung disease in acute OVA or chronic house dust mite (HDM) antigen exposure in *Nlrp12^−/−^* mice.

## Results

### NLRP12 does not affect OVA mediated allergic airway inflammation

**Figure 1:**
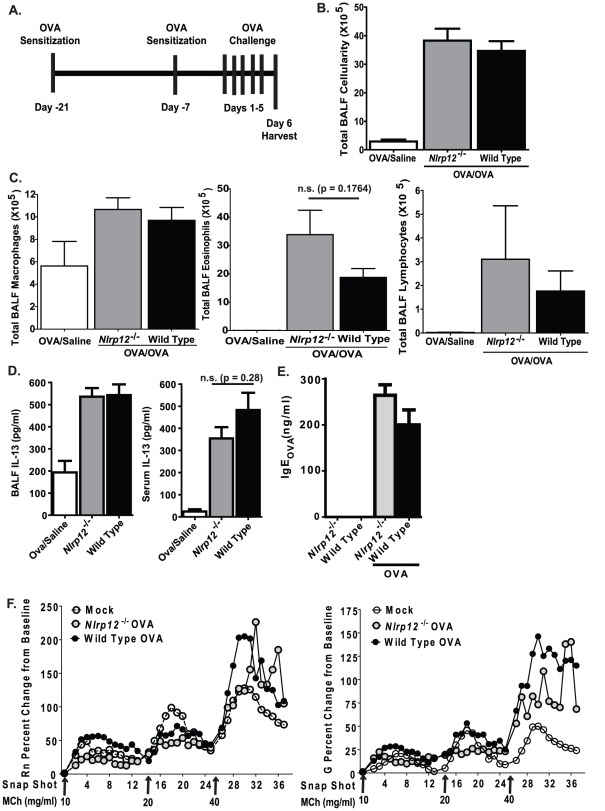
NLRP12 deficiency does not influence the development of acute allergic airway inflammation. **A**) Schematic illustrating OVA mediated acute allergic airway inflammation with an alum adjuvant. **B**) Mice immunized with OVA/alum and challenged with OVA (OVA/OVA) demonstrated a significant increase in BALF cellularity compared to wild type and *Nlrp12^−/−^* mice immunized with OVA/alum but challenged with saline (OVA/Saline). **C**) Differential staining of the BALF cellularity revealed that mice immunized and challenged with OVA demonstrated a significant increase in airway leukocyte populations, which were predominately eosinophilic. **D**) A significant increase in local (BALF) and systemic (serum) IL-13 was observed in all OVA immunized and challenged animals regardless of genotype. OVA/Saline, n = 6; *Nlrp12^−/−^*, n = 12; wild type, n = 15. **E**) A significant increase in OVA specific IgE was detected in serum collected from both wild type and *Nlrp12^−/−^* mice following OVA immunization and challenge. **F**) Central airway resistance (Rn) and tissue damping (G) in response to methacholine (MCh) was evaluated in wild type and *Nlrp12^−/−^* mice following OVA challenge. Mock (PBS), n = 4; Wild Type, n = 8; *Nlrp12^−/−^*, n = 5.

NLRP12 has been shown, *in vitro*, to attenuate signaling pathways that are essential mediators of the innate immune response to pathogens [5], [6]. However, recent data has demonstrated an *in vivo* role for NLRP12 in models of contact hypersensitivity [8]. *Nlrp12^−/−^* mice were found to have defective dendritic cell homing to the lymph node, which resulted in attenuated contact hypersensitivity to cutaneously applied allergens [8]. Therefore, we initially assessed the development of acute allergic airway inflammation in *Nlrp12^−/−^* mice. We utilized an ovalbumin (OVA) model, with aluminum hydroxide (alum) as an adjuvant during the sensitization stage, to induce airway inflammation (**Figure 1A**). OVA-mediated airway inflammation has long been utilized as a surrogate model for human asthma and has been useful in dissecting out major mediators of allergic airway inflammation and airway hyper-responsiveness. OVA sensitization followed by airway challenges with the antigen resulted in a significant increase in BALF cellularity in both *Nlrp12^−/−^* and wild type animals (**Figure 1B**). Morphological assessments of cells obtained from BALF revealed that the increased cellularity was associated with a significant increase in airway eosinophils and to a lesser extent increased macrophages and lymphocytes in both the *Nlrp12^−/−^* and wild type mice (**Figure 1C**). BALF from *Nlrp12^−/−^* show a trend towards enhanced eosinophils relative to wild type controls, but the difference was not statistically significant. These findings suggest that NLRP12 does not play a role in leukocyte migration into the airways in this model of allergic airway disease.

The interleukin IL-13 is a critical Th2 cytokine that drives the induction of airway inflammation, mucus production and airway hypersensitivity in the OVA model. Thus, we evaluated the role of NLRP12 in local and systemic production of IL-13 following the completion of the allergic airway disease model. As shown in **Figure 1D**, OVA sensitization and challenge resulted in a significant increase in BALF and serum levels of IL-13. However, no significant differences were observed between the *Nlrp12^−/−^* mice and wild type animals (**Figure 1D**). A previous study indicated that NLRP12 deficiency dramatically alters dendritic cell activity [8], which suggests that NLRP12 may influence antigen sensitization. Thus, in addition to IL-13, we also evaluated serum levels of immunoglobulin E (IgE). Following OVA immunization and challenge, we observed a significant increase in IgE levels. However, the levels of OVA specific IgE were not significantly altered between *Nlrp12^−/−^* and wild type mice (**Figure 1E**). Together, these data suggest that NLRP12 does not influence the generation of IL-13 or the production of antigen specific IgE during OVA mediated allergic airway disease.

Previous studies have noted an occasional disassociation between inflammation and airway hyperresponsiveness (AHR) in both human asthma and in mouse models. Thus, we also evaluated airway reactivity in the *Nlrp12^−/−^* mice. Following exposure to the bronchoconstricting agent methacholine (MCh), changes in central airway resistance (Rn) and tissue damping (G) were evaluated utilizing a small animal ventilator (flexiVent; Scireq). At the highest concentrations (40 mg/ml), MCh exposure generated a significant increase in both Rn and G in the OVA challenged mice (**Figure 1F**). However, no significant differences were observed between the wild type and Nlrp12−/− animals. Thus, NLRP12 deficiency does not alter the development of airway reactivity following OVA mediated airway inflammation.

**Figure 2:**
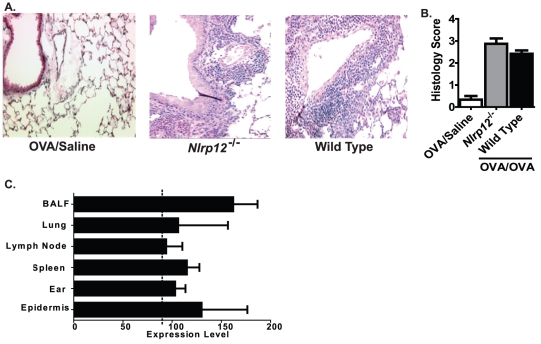
NLRP12 deficiency does not alter lung histopathology following the acute allergic airway inflammation model. Whole lungs were harvested 24 hours after the last airway challenge with either saline or OVA, and the left lobe was assessed and scored at specific locations along the main bronchi. Representative histology sections from the apical region along the main bronchi are shown. **A**) Lung inflammation was assessed in H&E stained sections (10× original magnification). **B**) Histology images were evaluated by blinded reviewers for a variety of inflammatory parameters and scored on a scale of 0 (absent) to 3 (severe). The scores for each parameter were averaged to generate the histology score as shown. A significant increase in inflammation was observed in mice immunized with OVA/alum and challenged with saline (OVA/Saline) compared to mice immunized with OVA/Alum but challenged with OVA (OVA/OVA). No significant differences in inflammation were observed between the *Nlrp12^−/−^* and wild type mice. OVA/Saline, n = 6; *Nlrp12^−/−^*, n = 12; wild type, n = 15. **C**) *Nlrp12* expression in mouse cells and tissues was compiled using a publically accessible microarray meta-analysis search engine (http://www.nextbio.com/b/search/ba.nb).

The OVA mediated allergic airway disease model is characterized by acute eosinophilic peribroncholar and perivascular inflammation. To further evaluate the role of NLRP12 in the resulting histopathology associated with this model, histological and morphometric assessments of lung sections were conducted. The following characteristics were evaluated in H&E stained lung sections: overall inflammation; perivascular and peribroncholar cuffing in the lung parenchyma and airway; and airway epithelial cell morphology (**Figure 2A**). As expected, lung inflammation was increased in all of the OVA sensitized and challenged mice (**Figure 2A**). However, as shown by the semi-quantitative histology score, no significant differences were detected in lung inflammation between the *Nlrp12^−/−^* mice and wild type animals (**Figure 2B**). Together, these data suggest that NLRP12 does not influence the induction of allergic airway inflammation in the acute OVA model.

NLRP12 was previously shown to influence the development of contact hypersensitivity [8]. Thus, we next considered the possibility that the lack of phenotype in the *Nlrp12^−/−^* mice could be associated with altered *Nlrp12* expression between the lung and the ear/epidermis. To evaluate this hypothesis, we assessed *Nlrp12* expression in relevant cells and tissues utilizing a publically accessible microarray meta-analysis search engine (http://www.nextbio.com/b/search/ba.nb). *Nlrp12* was expressed in cells isolated from BALF, lung, lymph node, spleen, ear and epidermis at comparable levels (**Figure 2C**). Thus, differences in *Nlrp12* expression are likely not associated with the differences in phenotype observed between the OVA mediated airway inflammation and contact hypersensitivity models.

**Figure 3:**
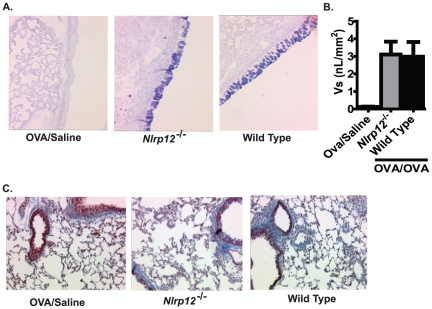
NLRP12 deficiency did not influence mucus production or collagen deposition following OVA sensitization and challenge. **A**) Mucus production was assessed following treatment with the Alcian blue/periodic acid-Schiffs reaction (AB/PAS) (20× original magnification). **B**) The volume of AB/PAS-stained mucosubstance per square millimeter of basal lamina (Vs) was determined. A significant increase in mucus was observed in the OVA/OVA mice; however, no significant differences were observed between the *Nlrp12^−/−^* and wild type mice. **C**) Masson's trichrome staining was used to assess collagen deposition in the lungs. No significant differences were observed for collagen deposition in the acute allergic airway inflammation model, regardless of genotype or treatment. OVA/Saline, n = 6; *Nlrp12^−/−^*, n = 12; wild type, n = 15.

In addition to airway inflammation, mucus hyperproduction and goblet cell hyperplasia and metaplasia is also a common finding in human asthmatics. Thus, we next sought to examine the contribution of NLRP12 in the production of airway mucus during the acute OVA model. Upon sensitization and challenge with OVA, both *Nlrp12^−/−^* and wild type mice displayed a significant increase in the number of goblet cells (**Figure 3A**). However, mucus quantification (Vs) using AB/PAS scoring did not reveal any significant differences in airway mucus hypersecretion between *Nlrp12^−/−^* and wild type mice (**Figure 3A–B**). This suggests that NLRP12 does not influence the production of mucus in the acute OVA model.

Subepithelial collagen deposition is a commonly observed feature of asthma and is typically associated with airway remodeling and hyper-responsiveness. The deposition of fibrin, collagen and collagen precursors is facilitated by a host of genes and many are regulated, either directly or indirectly by NF-κB. However, the airway remodeling that is typically observed in human asthma is not recapitulated in short term acute OVA mouse models. Because NLRP12 has been shown to function as a negative regulator of NF-κB, it is possible that loss of NLRP12 could result in accelerated airway remodeling. To evaluate this possibility, we utilized Masson's Trichrome staining of lung sections to assess collagen and collagen precursor deposition in the lungs. Consistent with the characteristics of this model, we did not observe an increase in Trichrome positive staining following the short term acute OVA airway challenge in any of the mice tested (**Figure 3C**). Thus, the deletion of *Nlrp12* did not appear to have an effect on collagen deposition in the OVA mediated allergic airway disease model.

### NLRP12 does not play a role in chronic, Dust Mite Allergen induced, allergic airway inflammation

**Figure 4:**
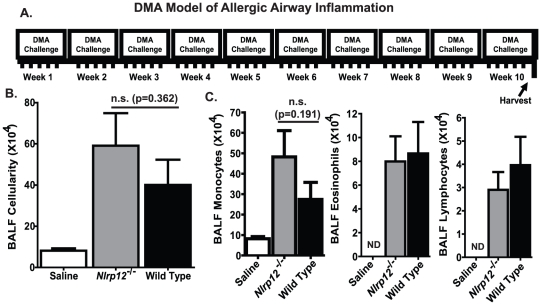
NLRP12 deficiency does not influence the development of Dust Mite Antigen induced chronic airway inflammation. **A**) Schematic illustrating the DMA mediated chronic airway inflammation model. **B**) Mice challenged with DMA demonstrated a significant increase in BALF cellularity compared to mice challenged with saline regardless of genotype. **C**) Differential staining of the BALF cellularity revealed that mice challenged with DMA demonstrated a significant increase in airway leukocyte populations, which were predominately monocytic in composition. Saline, n = 6; *Nlrp12^−/−^*, n = 8; wild type, n = 12.

The OVA/alum model is ideal for studying broad mechanisms associated with allergic airway disease. However, this model induces robust airway inflammation, which tends to obscure subtle phenotypes. Likewise, short term acute models such as the OVA/alum model utilized here do not accurately recapitulate many of the cardinal features associated with human asthma [10]. Therefore, we sought to assess the contribution of NLRP12 in a chronic model of allergic airway disease induced by house dust mite antigen (DMA) from *Dermatophagoides pteronyssinus* and *Dermatophagoides farinae* (**Figure 4A**). DMA is ubiquitous in the environment and has been shown to induce persistent Th2 driven airway inflammation and airway remodeling in mice. The clinical features associated with the DMA mouse model are more characteristic of human asthma than the OVA based acute airway disease models [11]. As seen in **Figure 4B**, chronic DMA administration induced a robust increase in BALF cellularity in both *Nlrp12^−/−^* and wild type mice. Further morphometric analysis revealed that the BALF cellularity was predominately composed of monocytes (**Figure 4C**). This is in sharp contrast to the predominant airway eosinophilia that is typically observed in the acute OVA based models. Eosinophils were significantly elevated in DMA treated animals (**Figure 4C**); however, this increase was minimal compared to the levels observed in the OVA treated animals (**Figure 1C**). No significant difference in total BALF cellularity or composition was detected between *Nlrp12^−/−^* and wild type mice.

**Figure 5:**
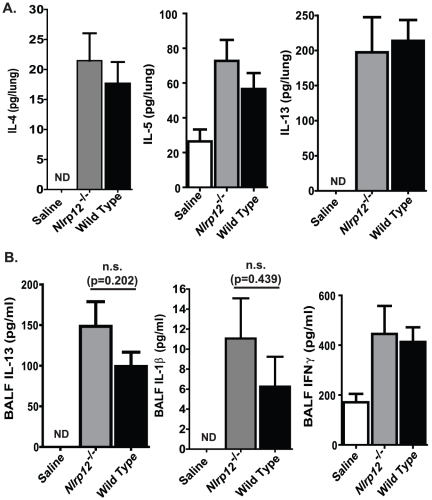
NLRP12 does not contribute to the production of Th2 associated cytokines following chronic HDM administration. **A–B**) A significant increase in Th2 associated proinflammatory mediators was observed in the lung (IL-4, IL-5, and IL-13), BALF (IL-13, IFN-γ) and serum (total IgE) from all DMA challenged animals. The Th1 associated cytokine, IL-1β, was also increased in the BALF; however, the data shown were extrapolated from the standard curve, which has 31.3 pg/ml as the lowest level of detection for the ELISA. Saline, n = 6; *Nlrp12^−/−^*, n = 8; wild type, n = 12.

Similar to the acute OVA model, DMA administration is also characterized by the induction of a wide array of Th2 cytokines and chemokines. In addition to IL-13, DMA has been reported to stimulate increased levels of IL-4 and IL-5, which are both critical for the production of allergen specific IgE and the recruitment of eosinophils to the airway. In the OVA models, the IL-4 and IL-5 can be difficult to accurately assess. Indeed, IL-4 and IL-5 were found to be below the level of detection in all of the samples assessed in the acute OVA model and in the serum and BALF from the mice treated with DMA (**data not shown**). However, as seen in **Figure 5A**, we were able to detect low levels of IL-4 and IL-5, and high levels of IL-13 in the supernatants collected from whole lung homogenates. DMA induced a significant increase in these cytokines in the lungs of all treated mice. However, no significant differences were detected in the cytokine levels between the *Nlrp12^−/−^* and wild type animals (**Figure 5A**). In addition to lung homogenates, we also assessed the BALF levels of IL-13, IL-1β and IFNγ. As seen in **Figure 5B**, we did observe a significant increase in IL-1β in the DMA treated animals. However, these levels were at the lower limits of detection for the ELISA kit. We also observed a significant increase in IFNγ in all mice that were challenged. However, no significant differences in IFNγ production was detected between the *Nlrp12^−/−^* and wild type mice.

**Figure 6:**
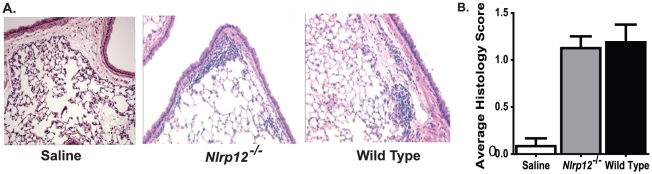
NLRP12 deficiency does not alter lung histopathology following chronic, DMA mediated, allergic airway inflammation. Whole lungs were harvested 24 hours after the last airway challenge with either saline or DMA, and the left lobe was assessed and scored at specific locations along the main bronchi. Representative histology sections from the apical region along the main bronchi are shown. **A**) Lung inflammation was assessed in H&E stained sections (10× original magnification). **B**) Histology images were evaluated by blinded reviewers for a variety of inflammatory parameters and scored on a scale of 0 (absent) to 3 (severe). The scores for each parameter were averaged to generate the histology score as shown. A significant increase in inflammation was observed in mice challenged with DMA compared to mice challenged with saline alone. No significant differences were observed between the *Nlrp12^−/−^* and wild type mice. Saline, n = 6; *Nlrp12^−/−^*, n = 8; wild type, n = 12.

**Figure 7:**
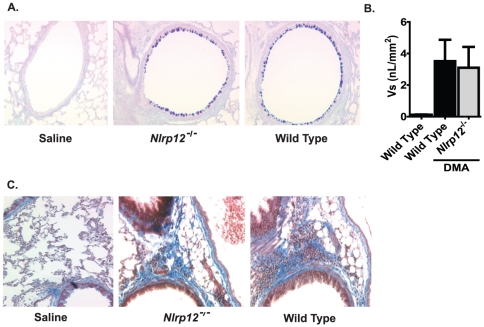
NLRP12 deficiency did not influence mucus production or collagen deposition in the lungs following HDM. **A**) Mucus production was assessed following treatment with the Alcian blue/periodic acid-Schiffs reaction (AB/PAS) (20× original magnification). **B**) The volume of AB/PAS-stained mucosubstance per square millimeter of basal lamina (Vs) was determined. A significant increase in mucus was observed in the DMA mice; however, no significant differences were observed between the *Nlrp12^−/−^* and wild type mice. **C**) Masson's trichrome staining was used to assess collagen deposition in the lungs. Histopathology assessments revealed an increase in collagen deposition in the DMA treated animals compared to the saline treated mice. No significant differences were observed between the *Nlrp12^−/−^* and wild type mice. Saline, n = 6; *Nlrp12^−/−^*, n = 8; wild type, n = 12.

Similar to OVA mediated allergic airway disease, chronic DMA induced allergic airway disease has been shown to induce significant increases in airway inflammation and mucus hyperproduction. However, unlike the acute nature of the OVA model, DMA induces a significantly attenuated disease. Likewise, because of the extended period of DMA exposure and chronic nature of the model, increased airway remodeling is a commonly identified feature. Following 10 weeks of i.n. DMA administration, we observed a significant increase in airway inflammation (**Figure 6A–B**) and airway mucus production (**Figure 7A–B**). Unlike the acute OVA model, we also observed an increase in Massons Trichrome positive regions in the lungs of mice treated with DMA (**Figure 7C**). Together, these data confirm previously reported accounts that the DMA model induces a less acute disease with clinical features that are more consistent with human asthma. However, in all cases, no significant differences were detected between *Nlrp12^−/−^* and wild type animals. Together, these data suggest that NLRP12 does not influence the development of either acute or chronic allergic airway disease.

## Discussion

NLRP12 has been shown to influence both canonical and non-canonical NF-κB signaling and classical and nonclassical MHC class I gene expression *in vitro* [5], [6], [12]. *In vivo*, *Nlrp12* deficiency significantly influences the development of contact hypersensitivity [8]. Based on these findings we hypothesized that the *Nlrp12^−/−^* mouse would provide a suitable model to study the contribution of this gene in allergic diseases, including asthma. However, despite notable trends, we did not detect any significant differences in the development of either acute OVA or HDM mediated allergic lung disease in the *Nlrp12^−/−^* mice compared to the wild type animals.

A number of explanations are possible that may explain our inability to observe significant differences in these lung disease models. The most obvious explanation is that NLRP12 does not have a discernable effect on the pathogenesis of allergic lung disease in the mouse or NLRP12 may function in either a temporal, situational or stimuli specific manner that was not captured in the data generated by the lung inflammation models discussed here. NLRP12 activity may be restricted to a specific cell type or tissue that may contribute to the development of contact hypersensitivity and atopic dermititis, while not significantly contributing to lung inflammation. This hypothesis is similar to *in vivo* data published for TLR2, which has been shown to play a vital role in the host immune response during contact hypersensitivity. Similar to NLRP12, TLR2 has been shown to be an essential mediator of the immune response to oxazolone (OX) in an allergic contact dermatitis model [13]. However, unlike the findings for OX, *Tlr2^−/−^* and wild type mice demonstrated similar levels of inflammation in models involving epicutaneous sensitization with OVA [13]. Other *in vivo* studies have suggested that TLR2 functions as a negative regulator of allergic airway inflammation following either DMA exposure or acute OVA challenges [14], [15], [16]. In the context of NLRP12, several studies have associated functional studies with gene expression data in humans and rodents. These studies have shown that *NLRP12* is differentially expressed between species and transiently increased during various models of lung inflammation [6], [8], [17]. Together, these data support a scenario where NLRP12 does not influence the development of allergic airway inflammation. However, as illustrated by the contact hypersensitivity phenotypes previously reported, it is likely that NLRP12 has a more dramatic role in other models of inflammatory diseases through a temporal and tissue specific mechanism.

A second hypothesis for the failure to observe an *in vivo* phenotype in the lung inflammation models in the *Nlrp12^−/−^* mice suggests that the models and analyses we utilized in this study are too broad to effectively discern mild or moderate phenotypes. Indeed, the OVA model described here induced a vigorous Th2 mediated immune response. One valid criticism of this model is that the acute nature and robust inflammation tends to obscure the contribution of several important mediators to the pathogenesis of the disease. To avoid this issue, studies have suggested utilizing chronic models, such as long term OVA exposure (without alum) or DMA exposure, to assess allergic airway inflammation [10], [11], [18]. Thus, in an effort to address this concern, we also utilized DMA to induce airway inflammation and did not observe any discernable phenotypic differences in the *Nlrp12^−/−^* mice. However, we cannot rule out the possibility that higher resolution *in vivo* models or analysis may reveal a more significant contribution for NLRP12 in mediating subtle aspects of inflammation in the lung.

While this study reports that NLRP12 does not affect detectable difference in allergic lung inflammation, it is important to recognize that additional *in vivo* assessments in the lung or in other tissues may reveal important functions for this NLR in other disease processes. Our data suggests that NLRP12 does not contribute in a detectable way to the development of allergic airway disease models tested here. However, it is interesting to speculate that it may function as either a positive or negative regulator of innate immune responses to agents associated with asthma exacerbations. Thus, our data shows that the acute OVA and chronic HDM models are appropriate for future studies that aim to decipher the contribution of NLRP12 in asthma exacerbations, which is an area of intense scientific and clinical interest.

## Materials and Methods

### Ethics Statement

All studies were conducted under the approval of the Institutional Care and Use Committee (IACUC) for The University of North Carolina at Chapel Hill (IACUC protocol approval #'s 07-170, 10-146, which are specific for the animal models utilized in this publication) and in accordance with the National Institutes of Health Guide for the Care and Use of Laboratory Animals.

### Experimental Animals

*Nlrp12^−/−^* animals were kindly provided by Millennium Inc. and were generated as previously described [8]. All animals were backcrossed 9 generations onto C57Bl/6 mice (Jackson Laboratories). Genotypes were confirmed via PCR of genomic tail DNA utilizing the following primer sets: Forward-1, 5′-CCCACAAAGTGATGTTGGACTG-3′, Forward-2, 5′-GCAGCGCATCGCCTT CTATC-3′, Reverse, 5′-GAAGCAACCTCCGAATCAGAC-3′. All mice were maintained under specific pathogen free conditions and all experiments were performed with 6–12 week old age- and sex- matched mice.

### Induction of allergic airway inflammation

Allergic airway inflammation was induced by ovalbumin (OVA) as previously described [19]. Mice were sensitized by i.p. injection of 20 µg of OVA (Grade V; Sigma) emulsified in aluminum hydroxide (Sigma) in a total volume of 200 µl, on days −21 and −7. Airway inflammation was induced via intranasal (i.n.) administration of OVA (1% in saline) for 5 days (days 1–5). Control mouse groups received the two OVA immunizations, but were i.n. challenged with saline. Mice were harvested 24 hours following the last i.n. administration of OVA.

Allergic airway inflammation (chronic) was induced by house dust mite antigen (DMA) challenge. Mice were exposed i.n. to 0.05 AU/ml of purified 50∶50 DerP and DerF whole body extract (Greer Laboratories, Lenoir, NC) in 50 ul of saline for 5 consecutive days, followed by 2 days of rest, for 10 consecutive weeks. Mice were harvested 24 hours following the last i.n. administration of DMA.

### Techniques for Assessing Airway Inflammation

Following the completion of the respective models, mice were euthanized and serum was collected by cardiac puncture. To assess the cellularity and cytokine levels in the bronchoalveolar lavage fluid (BALF), mice were perfused with HBSS and a tracheal cannula was inserted below the larynx. The lungs were lavaged 3 times with 1 ml of HBSS. The resultant BALF was centrifuged to separate the cellular components and cell free supernatants. Protein levels were assessed from serum, cell free lung homogenates and cell free BALF supernatants by ELISA (OptEIA, BD or R&D Biosystems). Total BALF cellularity was evaluated, following red blood cell lysis via hypotonic saline treatment, using a hemacytometer. BALF composition was determined by differential staining of samples that were cytospun onto slides and stained with Diff-Quik (Dade Behring). Leukocytes were characterized based on morphology assessments of ≥200 cells per BALF sample. Using morphology based assessments, all monocyte derived cells, including macrophages, were classified as monocytes.

For histopathology, following BALF collection, the lungs were fixed by inflation to 20-cm pressure, immersed in 4% paraformaldehyde (PFA) and whole inflated lungs were embedded in paraffin wax. To evaluate airway inflammation, fixed lung slices (5-µm) were stained with hematoxylin and eosin (H&E). Sections of the left lung lobe were cut to yield the maximum longitudinal visualization of the intrapulmonary main axial airway. These sections were examined and inflammation was scored by an experienced reviewer who was blinded to genotype and treatment. Histopathology was evaluated and the following inflammatory parameters were scored between 0 (absent) and 3 (severe): mononuclear and polymorphonuclear cell infiltration; airway epithelial cell hyperplasia and injury; extravasation; perivascular and peribroncheolar cuffing; and the percent of the lung involved with inflammation. This scoring system has been previously described [19], [20], [21]. These parameter scores were averaged for a total histology score. In addition to histopathology, goblet cell hyperplasia was also assessed in the allergic airway disease models. Sections of the left lung lobes were sectioned, as described above, and stained with the Alcian-blue/periodic acid-schiff reaction (AB/PAS). In an effort to avoid bias for certain regions and to consistently view the identical region in all slides, a 2-mm length of airway was marked and digitally imaged. The region that was evaluated is located midway along the length of the main axial airway. Using ImageJ software (NIH, National Technical Information Service, Springfield, VA), the length and area of the AB/PAS-stained region in the lung sections were measured and the data is expressed as the mean volume density (Vs = nl/mm2 basal lamina+SEM of AB/PAS-stained material within the epithelium), as previously described [22]. To evaluate collagen deposition in the lungs, sections were prepared as described above, stained with Masson's Trichrome and assessed by an experienced reviewer who was blinded to genotype and treatment, as previously descbribed [23].

### Measurement of Lung Function by Small Animal Ventilator

Mice were anesthetized with pentobarbital sodium, tracheostomized and paralyzed with pancuronium bromide. Mice were then mechanically ventilated with a computer controlled small animal ventilator (Scireq, Montreal, Canada) at 300 breaths/min, with a tidal volume of 6 cc/kg and a PEEP of 3–4 cm water. Mice were exposed via aerosol to challenges with 0, 10, 20 and 40 mg/ml of methacholine (MCh; Sigma, St. Louis), which was delivered by ultrasonic nebulizer (Scireq, Montreal, Canada) for 30 seconds. During the aerosol exposure, the ventilation rate was reduced to 200 breaths/min and 0.15 cc/kg tidal volume. Following the aerosol challenge, ventilation was resumed at the original rate. Forced Oscillatory Mechanics (FOM) were utilized to evaluate airway reactivity every 10 seconds for 3 minutes following each MCh challenge. Our analysis was focused on the evaluation of Newtonian Resistance (Rn), which reflects resistance in the central airways, and the tissue resistance (G).

### Statistical Analysis

All data are presented as the mean +/− the standard error of the mean (SEM). For complex data sets, we utilized an Analysis Of Variance (ANOVA) followed by either Tukey-Kramer HSD or Newman-Keuls for multiple comparisons. Single data points were assessed by the Student's two-tailed t-test. In all cases, a p-value of less than 0.05 was considered statistically significant.
